# Targeting LAG-3 and PD-1 to Enhance T Cell Activation by Antigen-Presenting Cells

**DOI:** 10.3389/fimmu.2018.00385

**Published:** 2018-02-27

**Authors:** Felix S. Lichtenegger, Maurine Rothe, Frauke M. Schnorfeil, Katrin Deiser, Christina Krupka, Christian Augsberger, Miriam Schlüter, Julia Neitz, Marion Subklewe

**Affiliations:** ^1^Department of Medicine III, University Hospital, LMU Munich, Munich, Germany; ^2^Laboratory for Translational Cancer Immunology, Gene Center, LMU Munich, Munich, Germany; ^3^German Cancer Consortium (DKTK) and German Cancer Research Center (DKFZ), Heidelberg, Germany

**Keywords:** cancer immunotherapy, dendritic cell, immune checkpoint molecules, LAG-3, PD-1, T cell response

## Abstract

Immune checkpoint inhibition has been shown to successfully reactivate endogenous T cell responses directed against tumor-associated antigens, resulting in significantly prolonged overall survival in patients with various tumor entities. For malignancies with low endogenous immune responses, this approach has not shown a clear clinical benefit so far. Therapeutic vaccination, particularly dendritic cell (DC) vaccination, is a strategy to induce T cell responses. Interaction of DCs and T cells is dependent on receptor–ligand interactions of various immune checkpoints. In this study, we analyzed the influence of blocking antibodies targeting programmed cell death protein 1 (PD-1), HVEM, CD244, TIM-3, and lymphocyte activation gene 3 (LAG-3) on the proliferation and cytokine secretion of T cells after stimulation with autologous TLR-matured DCs. In this context, we found that LAG-3 blockade resulted in superior T cell activation compared to inhibition of other pathways, including PD-1/PD-L1. This result was consistent across different methods to measure T cell stimulation (proliferation, IFN-γ secretion), various stimulatory antigens (viral and bacterial peptide pool, specific viral antigen, specific tumor antigen), and seen for both CD4^+^ and CD8^+^ T cells. Only under conditions with a weak antigenic stimulus, particularly when combining antigen presentation by peripheral blood mononuclear cells with low concentrations of peptides, we observed the highest T cell stimulation with dual blockade of LAG-3 and PD-1 blockade. We conclude that priming of novel immune responses can be strongly enhanced by blockade of LAG-3 or dual blockade of LAG-3 and PD-1, depending on the strength of the antigenic stimulus.

## Introduction

Immunotherapy has changed our approach to anti-cancer treatment in recent years. Checkpoint inhibitors have particularly been in the focus of clinical development and have shown remarkable success as monotherapy or as combination partners for various tumor entities. This has resulted in approval for different solid tumor entities, but also for Hodgkin lymphoma (1–4). Checkpoint blockade is thought to reactivate endogenous T cell responses directed against tumor neoantigens presented in the context of major histocompatibility complex (MHC) molecules. In tumors with low endogenous T cell responses, however, the primary goal of immunotherapy needs to be the initiation of T cell responses directed against tumor-associated antigens. Various vaccination concepts are being pursued, and only recently, personalized neoantigen-based vaccines were shown to efficiently trigger T cell responses and lead to improved clinical outcome in patients with malignant melanoma (5, 6).

Dendritic cells (DCs) are particularly eligible to induce strong and durable immune responses. Over the years, multiple different maturation protocols have been used to generate DCs from monocytes *ex vivo* (7), and the resulting DCs differ considerably in their immunostimulatory capacities. We have developed a GMP-compliant 3-day protocol for the generation of DCs with improved immunogenicity based on a toll-like receptor (TLR) 7/8 ligand (TLR-3-DCs) (8). These DCs express higher numbers of co-stimulatory molecules and secrete higher levels of IL-12p70 compared to DCs generated with the standard protocol (9). Currently, we are conducting a phase I/II study on vaccination with DCs loaded with Wilms Tumor 1 (WT1) and preferentially expressed antigen in melanoma as leukemia-associated antigens for postremission therapy of acute myeloid leukemia (AML) patients (10).

In order to further enhance immunological and clinical responses, multiple combinatorial approaches with DC vaccination can be considered. These include, but are not restricted to chemotherapy and radiotherapy, cytokines and TLR agonists, hypomethylating agents, but also more targeted strategies, such as elimination of immunosuppressive cell types (e.g., myeloid-derived suppressor cells, regulatory T cells), molecularly targeted therapies and adoptive cell therapy (11, 12).

Another promising approach is the combination of DC vaccination with immune checkpoint inhibitors (13). Activated or chronically stimulated T cells upregulate various co-inhibitory molecules, such as programmed cell death protein 1 (PD-1), CD244 (2B4), CD160, T-cell immunoglobulin and mucin-domain containing-3 (TIM-3, CD366), and lymphocyte activation gene 3 (LAG-3, CD223) (14, 15). Their ligands are expressed both on antigen-presenting cells (APCs) and tumor cells. The inhibition of these checkpoints by blocking antibodies can, thus, enhance a vaccination-induced anti-cancer immune response in two ways. On the one hand, checkpoint inhibitors influence the interaction between T cells and cancer cells, resulting in enhanced anti-cancer T cell responses. On the other hand, checkpoint blockade may enhance the antigen-specific activation of T cells by DCs or other APCs. Studies performed in this field so far mainly focus on the inhibition of the PD-1/PD-L1 pathway (16–21).

Other co-inhibitory molecules, however, are also expressed on APCs, even on DCs after maturation with a TLR ligand (9). We, therefore, analyzed the effects of blocking various immune checkpoints on the stimulation of T cells by autologous TLR-3-DCs, mainly using virus antigens as a model system. Besides PD-1, we tested HVEM, CD244, TIM-3, and particularly LAG-3.

LAG-3 is a member of the Ig superfamily that was identified in 1990 (22). It is structurally similar to CD4 and binds MHC class II with a higher affinity than CD4 (23, 24). LAG-3 is expressed on activated CD4^+^ and CD8^+^ T cells as well as on a subset of natural killer cells (22). By using a knock-out mouse model, LAG-3 was found to impede T cell expansion and to control the number of memory T cells (25). Besides effector cells, LAG-3 can also be found on the surface of T regulatory cells and seems to be instrumental for their suppressive activity (26) as well as for T cell homeostasis (27). Finally, LAG-3 is also expressed on plasmacytoid DCs (28). Thus, modulation of the LAG-3 pathway has the potential to impact autoimmunity and infections as well as cancer (29, 30). In three distinct transplantable tumor models, LAG-3 and PD-1 have been shown to be co-expressed on tumor-infiltrating lymphocytes, and blockade of both pathways had synergistic effects on the anti-tumor CD8^+^ T cell response (31). Similarly in ovarian cancer patients, co-expression of LAG-3 and PD-1 was found on antigen-specific CD8^+^ T cells, and co-blockade of both lead to improved proliferation and cytokine production (32). Accordingly, different LAG-3 antibodies as monotherapy or in combination with anti-PD-1 have entered clinical trials for various cancer entities focusing on solid tumors.

In our model, we found that priming of T cells by DCs is significantly enhanced by blockade of LAG-3. We, therefore, propose the combination of DC vaccination and LAG-3 blockade as a promising approach for the initiation of novel immune responses, particularly in tumors with low endogenous immune responses including AML.

## Materials and Methods

### Media and Reagents

Very low endotoxin RPMI 1640 medium (FG 1415; Biochrom) supplemented with 1.5% human serum (serum pool of AB positive adult males; Institute for Transfusion Medicine)—hereafter named DC medium—was used for the generation of DCs and all coculture experiments. The following reagents were used to generate DCs: GM-CSF (300-03), rhIL-4 (200-04), IFN-γ (300-02; all PeproTech), rhIL-1β (201-LB), TNF-α (210-TA/CF; both R&D Systems), PGE2 (P5640; Sigma-Aldrich), and R848 (tlrl-r848; InvivoGen).

### Cell Isolation and Generation of DCs

After written informed consent, peripheral blood (PB) samples were collected from healthy donors (HDs) under a clinical protocol entitled “*in vitro* studies to establish new immunotherapies for AML and other hematological neoplasias.” Both the consent form and the protocol were approved by the institutional review board (Ethikkommission bei der LMU München). Both cell isolation and generation of DCs were performed as described previously for TLR-3-DCs (9) with the exception of polyI:C, which was not included in the maturation cocktail.

### Coculture of DCs and T Cells

Dendritic cells were pulsed with a mixed CMV, EBV, influenza, and tetanus (CEFT) peptide pool (2 µg/ml; PM-CEFT; JPT) for 2 h at 37°C, 5% CO_2_, incubated for 10 min on ice and subsequently washed. CD3^+^ T cells were isolated from autologous non-adherent cells (NACs) by magnetic activated cell sorting (MACS, 130-050-101; Miltenyi Biotec) according to the manufacturer’s protocol. CEFT-pulsed DCs and CD3^+^ T cells were cocultured at a ratio of 1:10 in 96-well round bottom plates for 4 days at 37°C, 5% CO_2_. For blocking experiments, the following monoclonal blocking antibodies were added at 10 µg/ml: α-CD244 (PP35; 16-2449-81; eBioscience), α-HVEM (122; 318802), α-TIM-3 (F38-2E2; 345003), α-PD-1 (EH12.27H7; 329911; all BioLegend), α-LAG-3 (17B4; AG-20B-0012PF; AdipoGen or ab40466; Abcam). The blocking antibody concentration of 10 µg/ml that we used was based on prior experiments demonstrating antibody blockade of immune checkpoints (21). Reducing the antibody concentration to 5 µg/ml did not alter our results (data not shown).

### Coculture of DCs and NACs

Dendritic cells were pulsed with the Epstein–Barr nuclear Ag 3 A peptide FLRGRAYGL (FLR) (2 µg/ml; JPT) for 2 h at 37°C, 5% CO_2_ and subsequently washed. FLR-pulsed DCs and autologous NACs were cocultured at a ratio of 1:80 in 96-well round bottom plates for 6 days at 37°C, 5% CO_2_. For blocking experiments, α-PD-1 and α-LAG-3 were added as above.

### Culture of PBMCs

Peripheral blood mononuclear cells (PBMCs) were loaded with FLR and cultured in 96-well round bottom plates (5 × 10^5^/well) in the presence or absence of α-PD-1 and α-LAG-3 for 6–8 days at 37°C, 5% CO_2_.

### Surface Phenotyping of DCs and T Cells

Immunofluorescent staining of DC surface antigens was performed using a panel of fluorescence-conjugated monoclonal antibodies: CD80 (PE, L307.4; 560925), CD83 (APC, HB15e; 551073) CD86 (FITC, 2331 (FUN-1); 557343), CD273 (APC, MIH18; 557926), CD274 (FITC, MIH1; 558065; all BD Biosciences), Galectin-9 (PE, 9M1-3; 348906), CD48 (FITC, BJ40; 336706), HLA-DR (Pacific Blue, LN3; 327016; all BioLegend), HVEM (APC, 94801; FAB356A; R&D Systems). Corresponding isotype controls were used.

Immunofluorescent staining of T-cell surface antigens was performed using the following fluorescence-conjugated monoclonal antibodies: CD244 (PE, C1.7; 329507 or APC, C1.7; 329511), PD-1 (Brilliant Violet 421, EH12.7H7; 329919), CD3 (FITC, UCHT1; 300406), CD45RA (Brilliant Violet 421, HI100; 304129; all BioLegend), CD160 (APC, 688327; FAB6700A), TIM-3 (PE, 344823; FAB2365P; both R&D Systems), CD8 (PerCP-eFluor 710, SK1; 8046-0087; eBioscience), CD4 (APC-H7, RPA-T4; 560158; BD Biosciences), LAG-3 (ATTO 647N, 17B4; AG-20B-0012TS AdipoGen), CCR7 (CD197, APC, FR 11-11E8; 130-098-125; Miltenyi Biotec). Corresponding isotype controls were used. Intracellular FoxP3 staining was performed according to the manufacturer’s instructions (APC, 3G3; Miltenyi Biotec).

Cells were analyzed using a FACS LSR II (BD Biosciences). Post-acquisition analysis was performed using FlowJo software (version 9.7.6; Tree Star). The median fluorescence intensity (MFI) ratio was calculated by dividing the MFI of the measured population by the MFI of cells stained with the isotype-matched antibody. For the upregulation of checkpoint molecules, the percentage of positive cells (% positive) was obtained by setting the gate at or below 1% in the respective isotype control.

### Cytokine Secretion Measurement by Bead-Based Immunoassay

Secretion of IFN-γ and TNF-α was quantified by cytometric bead array (CBA) Flex Set (560111; BD Biosciences) according to the manufacturer’s instructions.

### CFSE Proliferation Assay

Isolated CD3^+^ T cells were labeled with carboxyfluorescein N-succinimidyl ester (CFSE, C34554; Life Technologies) and cultured in the presence of autologous DCs. Unstimulated T cells served as negative control. Harvested cells were then stained with antibodies for CD3 (APC, UCHT1; 300412; BioLegend), CD4 (APC-H7), and CD8 (PerCP-eFluor 710). The percentage of divided cells (% divided) was analyzed using FlowJo software.

### Fluorescence-Based Cell Sorting

Magnetic activated cell sorting-enriched CD3^+^ T cells were sorted according to CCR7 and CD45RA expression levels into naive T cells (T_naive_), central memory T cells (T_CM_), effector memory T cells (T_EM_), and effector memory RA T cells (T_EMRA_) using an Aria III (BD Biosciences).

### Expansion of WT1 Peptide-Specific T Cells

Wilms Tumor 1 antigen VLD (VLD = WT1 peptide VLDFAPPGA)-specific T cells were generated as previously described (33). Briefly, DCs were matured as described above. Autologous CD8^+^ T cells were isolated from NACs using the CD8^+^ T Cell Isolation Kit (130-096-495; Miltenyi Biotec) and incubated overnight in VLE-RPMI medium supplemented with 5% human serum and 5 ng/ml of IL-7 (200-07; Peprotech). DCs were pulsed with 2.5 µL/ml of the HLA-A*02:01-restricted VLD peptide (VLDFAPPGA; JPT) for 90 min and irradiated with 30 Gy. CD8^+^ T cells and DCs were cocultivated in a 4:1 T cell:DC ratio and incubated with 30 ng/ml of IL-21 (200-21; Peprotech) in the presence or absence of 10 µg/ml LAG-3 or PD-1 blocking antibodies for 72 h. On day 3, cocultures were expanded 1:1 by adding medium supplemented with 10 ng/ml IL-15 and IL-7 (200-07, 200-15; both Peprotech) and 10 µg/ml blocking antibodies. On days 6–7, cells were analyzed by flow cytometry using VLD multimer (WB3469; Immudex) and fluorescence-conjugated monoclonal antibodies (see above).

### Statistical Analysis

Data were analyzed using Prism 6 (GraphPad Software). All results are presented in box-and-whisker plots, with boxes representing the lower quartile, the median and the upper quartile, while the whiskers show the minimal and the maximal value. The significance of differences for pairwise comparison was determined using the two-tailed Wilcoxon signed rank test. *p* < 0.05 was considered statistically significant (* in all figures), while *p* < 0.01 is termed highly significant (** in all figures).

## Results

### TLR-3-DCs Expressed PD-L1 and HLA-DR

**Figure 1:**
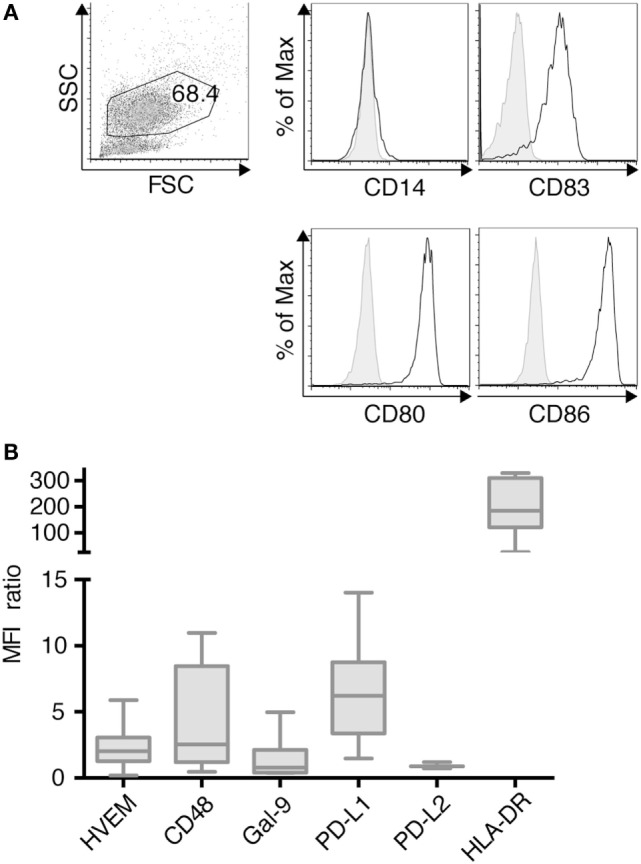
Immunophenotypic characterization of dendritic cells generated within 3 days based on a TLR7/8 ligand (TLR-3-DCs). TLR-3-DCs were generated from peripheral blood of healthy donor (HDs), and surface marker expression was measured by flow cytometry. **(A)** The characteristic phenotype of a dendritic cell population (FSChi/SSChi/CD14-/CD83+/CD80+/CD86+) is shown for one representative donor. **(B)** Expression of various inhibitory checkpoint molecules was analyzed on TLR-3-DCs of 3–10 donors, and MFI ratio of the expression is presented as box-and-whisker plots.

TLR-3-DCs were generated from PB of HDs. The characteristic phenotype of these DCs, with high expression of CD83, CD86, and CD80 and downregulation of CD14 is shown in Figure 1A. Expression of various inhibitory checkpoint molecules on DCs was analyzed by flow cytometry on 3–10 of these samples. HLA-DR was added to the panel as ligand for lymphocyte activation gene 3 (LAG-3) on T cells. MFI ratio of the expression data is presented in Figure 1B, statistical significance was tested against a theoretical median of 1.5. The expression of PD-L1 (median 6.2; *n* = 7; *p* = 0.004) and HLA-DR (median 184.5; *n* = 7; *p* = 0.016) on TLR-3-DCs was found to be (highly) significant. By contrast, HVEM (median 2.0; *n* = 10), CD48 (median 2.5; *n* = 7), Gal-9 (median 0.8; *n* = 7), and PD-L2 (median 0.9; *n* = 3) were not significantly expressed (Figure 1B).

### CD244, TIM-3, PD-1, and LAG-3 Were Upregulated on T Cells after Stimulation with TLR-3-DCs

**Figure 2:**
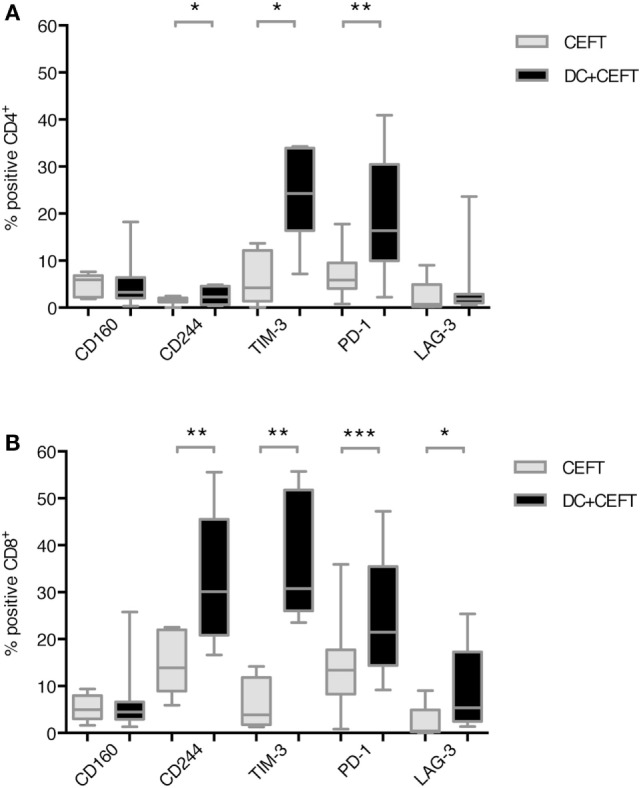
Upregulation of immune checkpoint ligands on T cells after dendritic cell stimulation. T cells of 7–14 healthy donor were cocultured with autologous TLR-3-DCs pulsed with CMV, EBV, influenza, tetanus (CEFT) peptide pool or with CEFT peptide pool alone. Expression of various inhibitory checkpoint molecules was analyzed by flow cytometry. The percentage of positive cells is presented as box-and-whisker plots for CD4^+^ **(A)** and for CD8^+^ **(B)** T cells. *p < 0.05; **p < 0.01.

Expression of the respective co-inhibitory ligands was determined on T cells with and without stimulation by DCs. TLR-3-DCs were generated from PB of HDs and pulsed with CEFT peptide pool. CD3^+^ T cells were isolated from PB of the same HDs and cocultured with autologous DCs or with CEFT peptide pool alone for 96 h. Expression of various inhibitory checkpoint molecules was analyzed on T cells by flow cytometry for 7–14 HDs. The percentage of positive cells is presented for CD4^+^ (Figure 2A; Figure S1 in Supplementary Material) and CD8^+^ (Figure 2B; Figure S1 in Supplementary Material) T cells. Statistical significance was tested between stimulation with pulsed DCs and CEFT stimulation alone as a control. CD4^+^ T cells showed a (highly) significant upregulation of CD244 (median of 2.3 vs. 1.5%; *n* = 7; *p* = 0.047), TIM-3 (median of 24.3 vs. 4.2%; *n* = 7; *p* = 0.016) and PD-1 (median of 16.4 vs. 5.9%; *n* = 13; *p* = 0.003) after stimulation with TLR-DCs, while expression of CD160 (median of 3.3 vs. 5.9%; *n* = 7) and LAG-3 (median of 1.8 vs. 0.7%; *n* = 9) were not changed (Figure 2A). On CD8^+^ T cells, we found (highly) significant upregulation of CD244 (median of 30.2 vs. 13.9%; *n* = 8; *p* = 0.008), TIM-3 (median of 30.8 vs. 3.9%; *n* = 8; *p* = 0.008), PD-1 (median of 21.5 vs. 13.4%; *n* = 14; *p* < 0.001) and LAG-3 (median of 5.4 vs. 0.4%; *n* = 9; *p* = 0.027), but not of CD160 (median of 4.5% vs. 5.0%; *n* = 8) (Figure 2B).

### Blockade of PD-1 and LAG-3, but Not HVEM, CD244 or TIM-3, Enhanced Proliferation of T Cells after Stimulation with TLR-3-DCs

**Figure 3:**
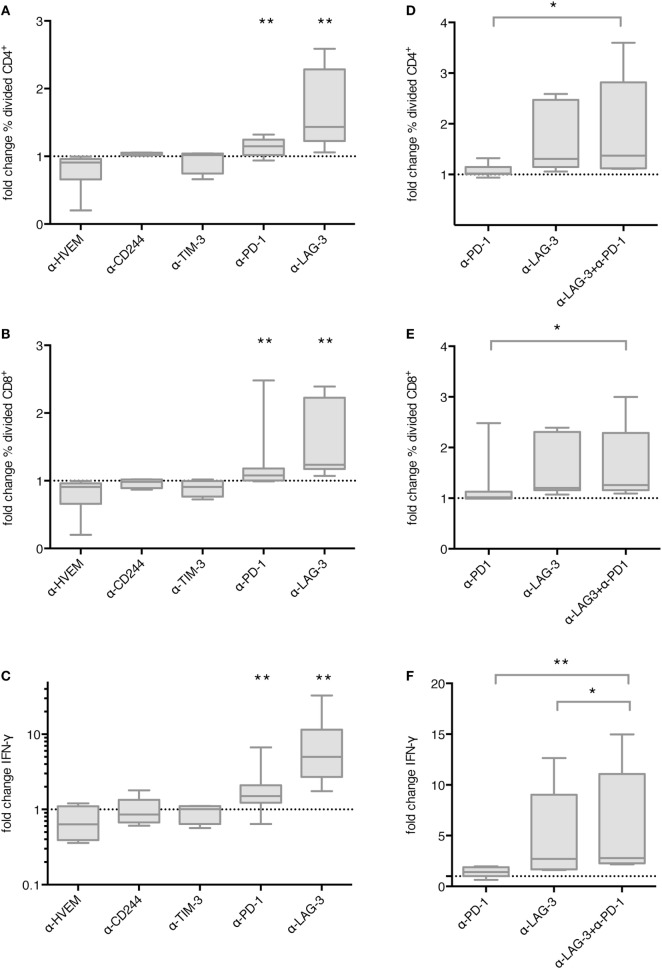
Effect of immune checkpoint blockade on proliferation and IFN-γ secretion of T cells after stimulation with TLR-3-DCs. CD3^+^ T cells of 4–14 healthy donor (HDs) were cocultured with autologous CMV, EBV, influenza, tetanus (CEFT)-pulsed TLR-3-DCs in the presence or absence of immune checkpoint blocking antibodies, either for individual antibodies **(A–C)** or in different combinations of α-PD-1 and α-LAG-3 antibodies **(D–F)**. Proliferation of CD4^+^ **(A,D)** and CD8^+^ T cells **(B,E)** was analyzed by carboxyfluorescein N-succinimidyl ester (CFSE) assay, and the ratio between the percentages of divided cells with and without blocking antibody was calculated. IFN-γ secretion of CD3^+^ T cells **(C,F)** was determined by cytometric bead array (CBA) assay, and the ratio between concentration with and without blocking antibody was calculated. All data are presented as box-and-whisker plots, and statistical significance was calculated against a fold change of 1.0. *p < 0.05; **p < 0.01.

In order to determine the functional relevance of co-inhibitory molecule interaction between TLR-3-DCs and T cells, we first tested the influence of checkpoint blockade on proliferation of T cells after DC stimulation. CD3^+^ T cells isolated from PB of HDs were labeled with CFSE and cocultured with autologous CEFT-pulsed TLR-3-DCs for 5 days in the presence or absence of respective blocking antibodies. The percentage of divided cells was determined by flow cytometry. The ratio between the percentages of divided cells with and without blocking antibody was calculated. Data for 4–13 samples is presented in Figure 3A for CD4^+^ T cells and in Figure 3B for CD8^+^ T cells, original data is shown in Table S1 in Supplementary Material. Statistical significance was calculated against a fold change of 1.0, equal to no effect of the blocking antibody on proliferation. For CD4^+^ T cells, no effect of checkpoint blockade on proliferation was found for HVEM (fold change 0.91; *n* = 6), CD244 (fold change 1.05; *n* = 4) and TIM-3 (fold change 1.02; *n* = 4). Blockade of PD-1 resulted in slightly enhanced proliferation (fold change 1.15; *n* = 13; *p* = 0.002), and blockade of LAG-3 lead to markedly enhanced proliferation (fold change 1.44; *n* = 9; *p* = 0.002), both statistically highly significant (Figure 3A). Similarly, for CD8^+^ T cells, blockade of PD-1 resulted in slightly enhanced proliferation (fold change 1.08; *n* = 13; *p* = 0.003), and blockade of LAG-3 lead to markedly enhanced proliferation (fold change 1.24; *n* = 9; *p* = 0.002), both statistically highly significant, while no effect of checkpoint blockade on proliferation was found for HVEM (fold change 0.88; *n* = 6), CD244 (fold change 0.96; *n* = 4) and TIM-3 (fold change 0.91; *n* = 4) (Figure 3B).

### Blockade of PD-1 and LAG-3, but Not HVEM, CD244 or TIM-3, Enhanced IFN-γ and TNF-α Secretion by T Cells after Stimulation with TLR-3-DCs

Next, we determined whether checkpoint blockade also influenced IFN-γ and TNF-α secretion by T cells after DC stimulation. CD3^+^ T cells isolated from PB of HDs were cocultured with autologous CEFT-pulsed TLR-3-DCs for 96 h in the presence or absence of respective blocking antibodies. The concentration of IFN-γ and TNF-α in the culture supernatant was determined by CBA. IFN-γ and TNF-α fold change was calculated by dividing the concentration of the coculture with blocking antibody by the concentration of the control coculture without antibody. Data for 5–14 samples is presented for IFN-γ in Figure 3C, statistical significance was calculated against a fold change of 1.0, original data are shown in Table S1 in Supplementary Material. No effect of checkpoint blockade on IFN-γ secretion was found for HVEM (fold change 0.63; *n* = 7), CD244 (fold change 0.86; *n* = 5), and TIM-3 (fold change 1.01; *n* = 5). Blockade of PD-1 resulted in enhanced IFN-γ secretion (fold change 1.50; *n* = 14; *p* = 0.002) and blockade of LAG-3 lead to markedly enhanced IFN-γ secretion (fold change 5.00; *n* = 9; *p* = 0.004), both statistically highly significant (Figure 3C). Similarly, no effect of checkpoint blockade on TNF-α secretion was found for HVEM (fold change 0.89; *n* = 7), CD244 (fold change 1.01; *n* = 5) and TIM-3 (fold change 0.92; *n* = 5), while blockade of PD-1 (fold change 1.69; *n* = 14; *p* = 0.002), and blockade of LAG-3 (fold change 5.29; *n* = 9; *p* = 0.008) resulted in enhanced TNF-α secretion, both statistically highly significant (Figure S2 in Supplementary Material).

### Combination with PD-1 Blockade Resulted in an Increase of IFN-γ Secretion, but Not in an Enhanced Proliferation of T Cells after Stimulation with TLR-3-DCs Compared to LAG-3 Blockade Alone

We tested the hypothesis that blockade of PD-1 and LAG-3 has additive or synergistic effects on proliferation or IFN-γ secretion by T cells after stimulation with TLR-3-DCs. For proliferation assays, CD3^+^ T cells isolated from PB of 7 HDs were labeled with CFSE and cocultured with autologous TLR-3-DCs for 5 days in the presence or absence of blocking antibodies for PD-1 and LAG-3, both alone and in combination. As above, the percentage of divided cells was determined by flow cytometry for the different conditions, and the ratio between the percentages of divided cells with and without blocking antibody was calculated. Data are presented in Figure 3D for CD4^+^ T cells and in Figure 3E for CD8^+^ T cells, statistical significance was calculated for the combination of blocking antibodies vs. single antibody blockade, original data are shown in Table S1 in Supplementary Material. For the combination of PD-1 and LAG-3 blockade (median fold change of 1.37 for CD4^+^ and 1.26 for CD8^+^), we found significantly higher T cell proliferation compared to PD-1 blockade alone (median fold change of 1.02 for CD4^+^; *p* = 0.016; 1.02 for CD8^+^; *p* = 0.016), but no difference to LAG-3 blockade alone (median fold change of 1.31 for CD4^+^; *p* = 0.094; 1.20 for CD8^+^; *p* = 0.250).

Similarly, for IFN-γ secretion assays, CD3^+^ T cells isolated from PB of 8 HDs were cocultured with autologous CEFT-pulsed TLR-3-DCs for 96 h in the presence or absence of blocking antibodies for PD-1 and LAG-3, both alone and in combination. The concentration of IFN-γ in the culture supernatant was determined by CBA. IFN-γ fold change was calculated as a ratio between the IFN-γ concentration of the coculture with and without blocking antibody. Statistical significance was calculated for the combination of blocking antibodies vs. single antibody blockade. For the combination of PD-1 and LAG-3 blockade (median fold change of 2.80), the increase in IFN-γ secretion compared to PD-1 blockade alone was statistically highly significant (median fold change of 1.41; *p* = 0.008). In comparison to LAG-3 blockade alone, we found a slight, but statistically significant enhancement (median fold change of 2.70; *p* = 0.016) (Figure 3F). Taken together, LAG-3 blockade alone resulted in strong enhancement of T cell proliferation and IFN-γ secretion. The effect on IFN-γ secretion was slightly increased by the combination with PD-1 blockade, while no additional effect was seen for T cell proliferation.

### LAG-3 Blockade Mainly Enhanced IFN-γ Secretion by Naive and T_CM_, While PD-1 Blockade Also Resulted in an Increase of IFN-γ Secretion by Effector Memory Cells

**Figure 4:**
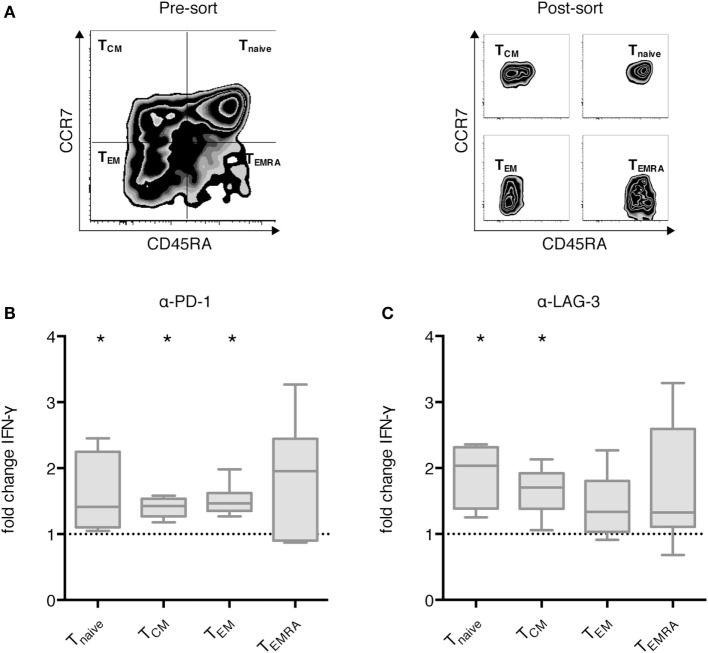
Effect of programmed cell death protein 1 (PD-1) and lymphocyte activation gene 3 (LAG-3) blockade on IFN-γ secretion of different T cell subpopulations after stimulation with TLR-3-DCs. MACS-enriched CD3^+^ T cells of 8 healthy donor (HDs) were sorted according to CCR7 and CD45 RA expression **(A)**. The various T cell subpopulations were cocultured with autologous CMV, EBV, influenza, tetanus (CEFT)-pulsed TLR-3-DCs in the presence or absence of α-PD-1 **(B)** and α-LAG-3 **(C)** antibody. IFN-γ secretion was determined by cytometric bead array (CBA) assay, and the ratio between concentration with and without blocking antibody was calculated. All data are presented as box-and-whisker plots, and statistical significance was calculated against a fold change of 1.0. *p < 0.05.

Next, we analyzed the differential effect of PD-1 and LAG-3 blockade on T cell subpopulations. MACS-enriched CD3^+^ T cells were sorted according to CCR7 and CD45RA expression levels into T_naive_, T_CM_, T_EM_, and T_EMRA_ T cells (Figure 4A). The various T cell populations were cocultured with autologous CEFT-pulsed TLR-3-DCs for 96 h in the absence of presence of blocking antibodies for PD-1 and LAG-3. Again, the concentration of IFN-γ in the culture supernatant was determined by CBA. IFN-γ fold change was calculated by dividing the concentration of the coculture with blocking antibody by the concentration of the control coculture without antibody. Data for six samples is presented for PD-1 (Figure 4B) and for LAG-3 (Figure 4C) blockade. Statistical significance was calculated against a fold change of 1.0. We found that PD-1 blockade lead to significantly increased IFN-γ secretion of T_naive_ (median fold change of 1.41; *p* = 0.031), T_CM_ (median fold change of 1.43; *p* = 0.031), and T_EM_ (median fold change of 1.47; *p* = 0.031), while the increased secretion of T_EMRA_ was not statistically significant (median fold change of 1.96; *p* = 0.156) (Figure 4B). By contrast, LAG-3 blockade had significant effects on IFN-γ secretion of T_naive_ (median fold change of 2.04; *p* = 0.031) and T_CM_ (median fold change of 1.71; *p* = 0.031), but not on T_EM_ (median fold change of 1.34; *p* = 0.094) and T_EMRA_ (median fold change of 1.33; *p* = 0.094) (Figure 4C). With respect to the CD25^+^/FoxP3^+^ regulatory T cell subpopulation of CD4^+^ T cells, we saw a tendency toward a higher percentage after LAG-3 blockade (Figure S3 in Supplementary Material).

### Blockade of LAG-3, but Not PD-1, Enhanced Proliferation of EBV Antigen-Specific T Cells after Stimulation with TLR-3-DCs

**Figure 5:**
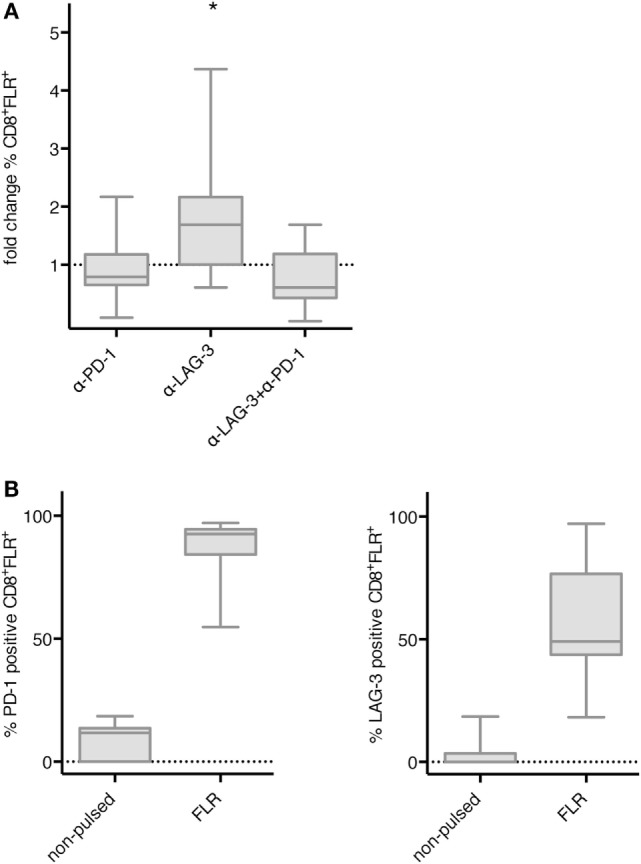
Effect of programmed cell death protein 1 (PD-1) and lymphocyte activation gene 3 (LAG-3) blockade on proliferation of EBV antigen-specific T cells after stimulation with TLR-3-DCs. non-adherent cell (NACs) of 9 healthy donor (HDs) were cocultured with autologous Epstein–Barr nuclear Ag 3 A peptide FLRGRAYGL (FLR)-pulsed TLR-3-DCs in the presence or absence of α-PD-1 and α-LAG-3 antibody. **(A)** The percentage of FLR tetramer positive cells within the CD8^+^ T cell population was determined by flow cytometry. Data for fold change to the condition without blocking antibody are presented as box-and-whisker plots, and statistical significance was calculated against a fold change of 1.0. *p < 0.05. **(B)** PD-1 and LAG-3 expression was determined for FLR tetramer positive CD8^+^ T cells after stimulation with non-pulsed or FLR-pulsed TLR-3-DCs.

Next, we tested whether blockade of PD-1 and LAG-3 also enhances the proliferation of antigen-specific T cells after stimulation with TLR-3-DCs. NACs (mainly consisting of T cells) of 9 HDs were cocultured with autologous FLR-pulsed TLR-3-DCs for 144 h in the presence or absence of blocking antibodies for PD-1 and LAG-3, both alone and in combination. The percentage of FLR tetramer positive (Tet^+^) cells within the CD8^+^ T cell population was determined by flow cytometry. Tet^+^ fold change was calculated by dividing the percentage in the condition with blocking antibody by the percentage in the condition without any antibody. Statistical significance was calculated against a fold change of 1.0. Blockade of LAG-3 resulted in a significantly increased percentage of Tet^+^ CD8^+^ T cells (median fold change 1.69; *p* = 0.039), while blockade of PD-1 (median fold change 0.79) and the combination of LAG-3 and PD-1 blockade (median fold change 0.61) did not enhance the percentage of antigen-specific T cells (Figure 5A). This was not due to a lack of PD-1 expression on T cells, as further analysis of the Tet^+^ CD8^+^ T cells after stimulation with FLR-pulsed DCs revealed that PD-1 was expressed on 92.6% of the T cells, while LAG-3 was found on only 49.1% of T cells (Figure 5B).

### Blockade of LAG-3, but Not PD-1, Enhanced Proliferation and IFN-γ Secretion of T Cells after Stimulation with FLR-Pulsed APCs within PBMCs

**Figure 6:**
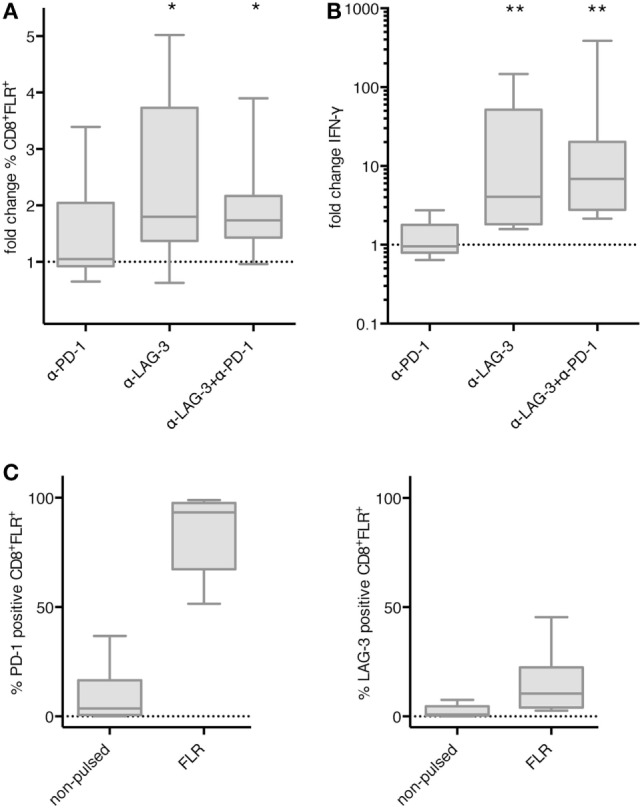
Effect of programmed cell death protein 1 (PD-1) and lymphocyte activation gene 3 (LAG-3) blockade on proliferation and IFN-γ secretion of EBV antigen-specific T cells after stimulation with antigen-presenting cells (APC) within peripheral blood mononuclear cells (PBMCs). Epstein–Barr nuclear Ag 3 A peptide FLRGRAYGL (FLR) peptide-pulsed PBMCs of 8 healthy donor (HDs) were cultered in the presence or absence of α-PD-1 and α-LAG-3 antibody. The percentage of FLR tetramer positive cells within the CD8^+^ T cell population was determined by flow cytometry **(A)**, and IFN-γ secretion was determined by cytometric bead array (CBA) assay **(B)**. Data for fold change to the condition without blocking antibody are presented as box-and-whisker plots, and statistical significance was calculated against a fold change of 1.0. * *p* < 0.05; **p < 0.01. **(C)** PD-1 and LAG-3 expression was determined for FLR tetramer positive CD8^+^ T cells after stimulation with non-pulsed or FLR-pulsed PBMCs.

We then asked if the effect of LAG-3 blockade on proliferation and IFN-γ secretion also holds true, if T cells are not stimulated by TLR-3-DCs, but by the various APCs naturally occurring within PBMCs. PBMCs of 8 HDs were pulsed with FLR peptide and cultured for 6 days in the presence or absence of blocking antibodies for PD-1 and LAG-3, both alone and in combination. Thereafter, the percentage of FLR tetramer positive cells (Tet^+^) within the CD8^+^ T cell population was determined by flow cytometry. Tet^+^ fold change was calculated by dividing the percentage in the condition with blocking antibody by the percentage in the condition without any antibody. Statistical significance was calculated against a fold change of 1.0. Blockade of LAG-3 resulted in a significantly increased percentage of Tet^+^ CD8^+^ T cells (median fold change 1.80; *p* = 0.023), while blockade of PD-1 (median fold change 1.05) did not enhance the percentage of antigen-specific T cells. The combination of LAG-3 and PD-1 blockade also significantly enhanced the percentage of Tet^+^ CD8^+^ T cells (median fold change 1.74; *p* = 0.016), but this was not different from LAG-3 alone (*p* = 0.461) (Figure 6A).

The concentration of IFN-γ was determined in the culture supernatant after 6–8 days of coculture by CBA. IFN-γ fold change was calculated by dividing the concentration of the coculture with blocking antibody by the concentration of the control coculture without antibody. Data for the same eight samples is presented in Figure 6B, statistical significance was calculated against a fold change of 1.0. Blockade of PD-1 (median fold change 0.96) did not enhance IFN-γ secretion, while increase of IFN-γ secretion after blockade of LAG-3 was highly significant (median fold change 4.07; *p* = 0.008). The combination of LAG-3 and PD-1 blockade also enhanced IFN-γ secretion highly significantly (median fold change 6.88; *p* = 0.008), but the difference to LAG-3 blockade alone was not significant (*p* = 0.188) (Figure 6B).

Further analysis of the Tet^+^ CD8^+^ T cells after stimulation with FLR-pulsed PBMCs revealed that PD-1 was expressed on almost all of the T cells (93.3%), while LAG-3 was found on only 10.5% of T cells (Figure 6C). Therefore, the non-existent effect of PD-1 blockade in this setting was not due to an absence of PD-1 on the T cell surface.

### Blockade of LAG-3, More than PD-1, Enhanced Expansion of WT1 Tumor Antigen-Specific T Cells after Stimulation with TLR-3-DCs

**Figure 7:**
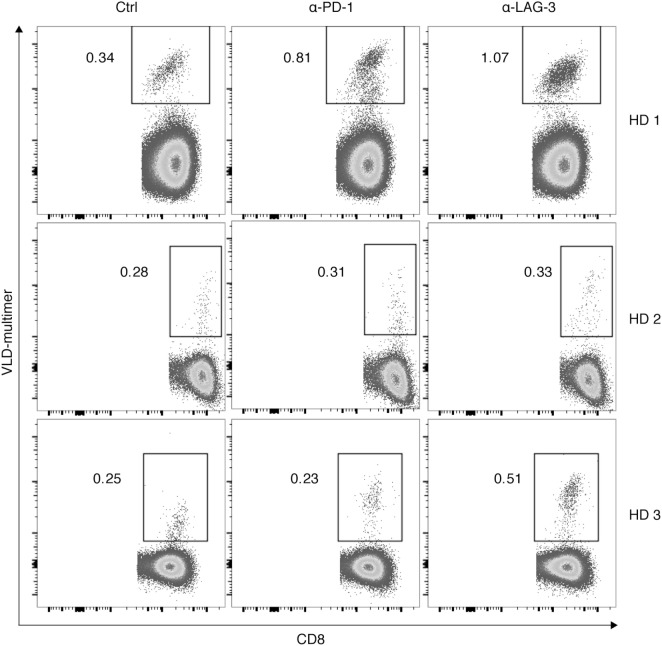
Effect of programmed cell death protein 1 (PD-1) and lymphocyte activation gene 3 (LAG-3) blockade on proliferation of Wilms Tumor 1 tumor-antigen-specific T cells after stimulation with TLR-3-DCs. CD8^+^ T cells of three healthy donor (HDs) were cocultured with autologous VLD-pulsed. TLR-3-DCs in the presence or absence of α-PD-1 and α-LAG-3 antibody. The percentage of VLD tetramer positive cells within the CD8^+^ T cell population was determined by flow cytometry. Data for all three donors are shown.

Finally, we tested the hypothesis that the effect of LAG-3 blockade on proliferation of antigen-specific T cells can also be transferred to tumor antigen specificity. CD8^+^ T cells of 3 HDs were cocultured with autologous TLR-3-DCs pulsed with a WT1 antigen (VLD peptide) for 6–7 days in the presence or absence of blocking antibodies for PD-1 and LAG-3. The percentage of VLD tetramer positive (Tet^+^) cells within the CD8^+^ T cell population was determined by flow cytometry. Results for all three donors are presented in Figure 7. Blockade of LAG-3 resulted in an increased percentage of Tet^+^ CD8^+^ T cells in two of three cases, while blockade of PD-1 resulted in an increase in Tet^+^ CD8^+^ T cells in only one case, and to a lesser extent.

## Discussion

Over the last decades, DCs generated *in vitro* for the vaccination of tumor patients have been optimized with respect to cytokine production as well as co-stimulatory molecule expression. However, even TLR-3-DCs, which demonstrate an improved phenotype and functional profile, express co-inhibitory molecules (9). Combining DC vaccination with checkpoint inhibition is, therefore, conceivable and might enhance T cell responses.

In this study, we systematically analyzed the effect of different checkpoint inhibitors on T cell stimulation by TLR-3-DCs. We found that within our experimental settings, blockade of LAG-3 was consistently superior to PD-1 blockade, independently of the method to measure T cell stimulation (proliferation, IFN-γ secretion), the stimulating antigen (viral and bacterial peptide pool, specific viral antigen, specific tumor antigen), and the type of T cell (CD4^+^ and CD8^+^ T cells) used. This was not expected, as LAG-3 expression on T cells is relatively low compared to PD-1 expression and only slightly upregulated after stimulation. However, it has to be considered that checkpoint molecules are often upregulated on antigen-specific T cells only (Figure S4A in Supplementary Material) and, thus, the assessment of bulk T cell populations might only insufficiently reflect checkpoint molecule expression. Besides, HLA-DR as the main ligand for LAG-3 is much higher expressed on APCs including TLR-3-DCs than any co-inhibitory molecules (Figure 1), conceivably resulting in numerous receptor–ligand interactions with T cells that help to explain the strong effects seen in our blocking experiments. LSECtin, a cell surface lectin of the DC-SIGN family, has been identified as an alternative ligand for LAG-3, and LAG-3 blockade has been shown to result in abrogation of immunoinhibitory effects of LSECtin in a melanoma mouse model (34). As LSECtin is only marginally expressed on TLR-3-DCs (Figure S5 in Supplementary Material), the effects of LAG-3 blockade demonstrated here are more likely due to interaction with HLA-DR. Similarly, the low PD-L2 expression on DCs suggests that PD-L1 is more relevant for the interaction with PD-1 in our setting. However, we cannot rule out that other receptor–ligand interactions between DCs and T cells that have not yet been explored are responsible for the effects on T cell responses that we describe. In order to further elucidate the mechanism of action, a potential approach could be the application of MHC class II blocking antibodies. In a model using COS-7 cells transfected with human LAG-3 and MHC class II-expressing human B lymphoblastic cell lines, it could be shown that both blocking antibodies against LAG-3 and HLA-DR were able to disrupt the rosettes formed by these cells (23). However, the exact binding site on MHC class II for LAG-3 is still unknown making the choice of an antibody that specifically blocks the interaction of MHC II with LAG-3 technically challenging.

In a recently published study that analyzed the effects of checkpoint blockade on T cell stimulation by allogeneic DCs, the addition of an antibody directed against LAG-3 to the coculture did not result in significant changes in T cell proliferation or cytokine secretion (21). As the setting of these experiments differed from ours in the origin of the blood donors (allogeneic vs. autologous), maturation protocol of the DCs and target antigens, there are multiple reasons for the diverging results. However, it is also important to notice that the LAG-3 antibody used is of a different clone and its blockade of the ligand–receptor interactions might be less effective than in our experiments. While we did not directly proof that the antibodies we used were blocking the interaction with their ligands, we only chose antibodies that had been described in the literature to have this capacity. Besides, we showed that addition of the blocking antibodies reduced the capacity of the respective staining antibody to bind to the receptor (Figure S4B in Supplementary Material).

While the effects of PD-1 blockade on T cell stimulation by TLR-3-DCs were less pronounced than those of LAG-3 blockade in our experiments, they were still significant. Surprisingly, however, the combination of both blocking antibodies did not result in a relevant increase in T cell stimulation compared to the LAG-3 antibody alone. In the analysis of viral antigen-specific T cell stimulation, it was even deleterious (Figure 5). Several murine tumor models, including a B16 melanoma and an MC38 colon adenocarcinoma model (31) demonstrated synergistic anti-tumor immunity by dual blockade of PD-1 and LAG-3. One possible explanation for our observation is an overstimulation of T cells by the combination of the immunostimulatory TLR-3-DCs with two effective checkpoint inhibitors. This is in line with data published for chronic lymphocytic leukemia, where PD-1 blockade abolished the positive effect induced by anti-LAG-3 antibodies in combination with CD3/CD28 beads as a very strong stimulus (35).

This hypothesis was substantiated in our experiments using PBMCs, comprising APCs that are relatively less immunostimulatory compared to TLR-3-DCs. Here, the combination of both blocking antibodies resulted in T cell stimulation that was at least similar to the LAG-3 antibody alone (Figure 6). As the strength of the antigen stimulus is also dependent on peptide concentration, we conducted peptide titration assays in the setting of viral antigen-specific T cell stimulation both by TLR-3-DCs and by PBMCs (Figure S6 in Supplementary Material). At the lowest peptide concentration, the combinatorial blockade was equally effective to LAG-3 blockade alone for DCs, while the effect of LAG-3 blockade on PBMCs was strongly increased by the addition of PD-1 blockade. Thus, we provide evidence that LAG-3 blockade alone is effective in boosting of T cell stimulation by a strong antigenic stimulus, while the combination of LAG-3 and PD-1 blockade is more effective in the setting of weak T cell stimulation. This observation is in line with *ex vivo* T cell stimulation experiments with tumor-infiltrating lymphocytes of epithelial ovarian cancer patients, where dual blockade of LAG-3 and PD-1 during priming of tumor antigen-specific T cells with tumor-derived APCs as weak stimulators increased T cell effector function to the levels observed with PB-derived APCs as stronger stimulators (32).

Our data set is focused on the priming phase of the immune response rather than the effector phase. The expression levels of checkpoint molecules on APCs clearly differ from those on tumor cells. Therefore, it is not surprising that the dominant effect of LAG-3 blockade and the relatively low effect of PD-1 blockade that we see deviates from the results in animal studies (36) and the outstanding clinical effects observed with PD-1 blockade as monotherapy or in combination with antineoplastic agents in clinical trials for various tumor entities. The effects observed in those studies rely on the effector phase of the immune response and are dependent on pre-existing effector T cells. Different immune checkpoints seem to be of importance in priming and effector phase, as directly shown for the epithelial ovarian cancer model, where LAG-3 blockade did not influence the effector function of already primed tumor-infiltrating T cells (32). Similarly, a 4-1BB agonist was more effective than an anti-LAG-3 blocking antibody as a combination partner for PD-1 blockade in a melanoma mouse model in the absence of any cancer vaccine (37). Recently, first data was published from an ongoing clinical trial (NCT01968109), in which anti-LAG-3 in combination with anti-PD-1 showed activity in melanoma patients who were relapsed or refractory to anti-PD-1/-PD-L1 therapy. The objective response rate (ORR) was 11.5% in 61 efficacy-evaluable patients, and a correlation of higher ORR with a LAG-3 expression above 1% on tumor-associated immune cells was shown (38).

Checkpoint blockade has revolutionized cancer therapy in several entities, including melanoma, lung cancer, and urothelial carcinoma. To our current understanding, these results primarily rely on reversing adaptive immune escape mechanisms of the tumor cells in the context of an immune response. Our data, however, support the relevance of checkpoint inhibition within the induction of primary or secondary anti-tumor immune responses. Thus, checkpoint inhibitors might also be therapeutically beneficial in tumor entities with a non-immunogenic microenvironment. Further studies will be needed to address the question of checkpoint inhibition within the priming versus effector phase of T cell responses. The sequencing and exact timing of LAG-3 and PD-1 blockade might be of particular relevance for the induction for optimal anti-tumor T cell responses.

## Author Contributions

FL, MR and MS conceived and designed the experiments. MR, FS, KD, CK, CA, MS, and JN performed the experiments. FL, MR, and MS analyzed the data and designed the figures. FL and MS wrote the manuscript. All authors read and approved the final manuscript.

## Conflict of Interest Statement

The authors declare that the research was conducted in the absence of any commercial or financial relationships that could be construed as a potential conflict of interest.

## Abbreviations

AML, acute myeloid leukemia; APC, antigen-presenting cell; CBA, cytometric bead array; CEFT, CMV, EBV, influenza, tetanus; CFSE, carboxyfluorescein N-succinimidyl ester; DC, dendritic cell; FLR, Epstein–Barr nuclear Ag 3 A peptide FLRGRAYGL; HD, healthy donor; LAG-3, lymphocyte activation gene 3; MACS, magnetic activated cell sorting; MFI, median fluorescence intensity; MHC, major histocompatibility complex; NAC, non-adherent cell; PB, peripheral blood; PBMC, peripheral blood mononuclear cell; PD-1, programmed cell death protein 1; PRAME, preferentially expressed antigen in melanoma; ORR, objective response rate; T_CM_, central memory T cell; T_EM_, effector memory T cell; T_EMRA_, effector memory RA T cell; T_naive_, naive T cell; TIM-3, T-cell immunoglobulin and mucin-domain containing-3; TLR, toll-like receptor; TLR-3-DCs, dendritic cells generated within 3 days based on a TLR7/8 ligand; VLD, WT1 peptide VLDFAPPGA; WT1, Wilms Tumor 1.
