# Supplementary material for: Targeting LAG-3 and PD-1 to Enhance T Cell Activation by Antigen-Presenting Cells
Source: Front Immunol. 2018 Feb 27;9:385. doi: 10.3389/fimmu.2018.00385 (PMC5835137; doi:10.3389/fimmu.2018.00385)
Supplement: Supplementary file 1 [file Data_Sheet_1.PDF]

Supplementary Material

Targeting LAG-3 and PD-1 to Enhance T Cell Activation by Antigen-Presenting Cells

Felix S. Lichtenegger[1,2§], Maurine Rothe[1,2§], Frauke M. Schnorfeil[1,2], Katrin Deiser[1,2], Christina Krupka[1,2], Christian Augsberger[1,2], Miriam Schlüter[1,2], Julia Neitz[1,2] and Marion Subklewe[1,2,3*]

[1]Department of Medicine III, University Hospital, LMU Munich, Germany

[2] Laboratory for Translational Cancer Immunology, Gene Center, LMU Munich, Germany

[3] German Cancer Consortium (DKTK) and German Cancer Research Center (DKFZ), Heidelberg, Germany

[§] These authors contributed equally.

*** Correspondence:**

Marion.Subklewe@med.uni-muenchen.de

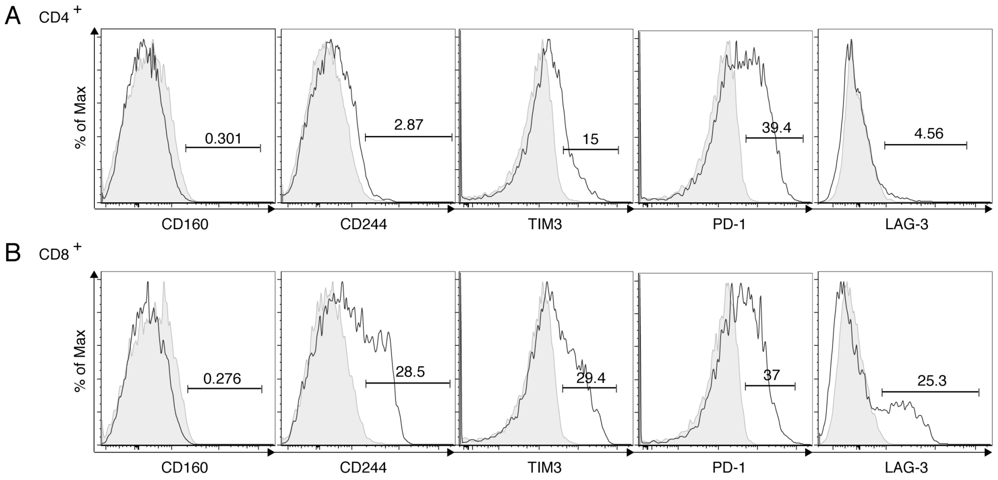

Figure S1. Upregulation of immune checkpoint ligands on T cells after DC stimulation. T cells were cocultured with autologous TLR-3-DCs pulsed with CEFT peptide pool. Expression of various inhibitory checkpoint molecules after 4 days of coculture was analyzed by flow cytometry. Original histogram data are shown for one representative donor.

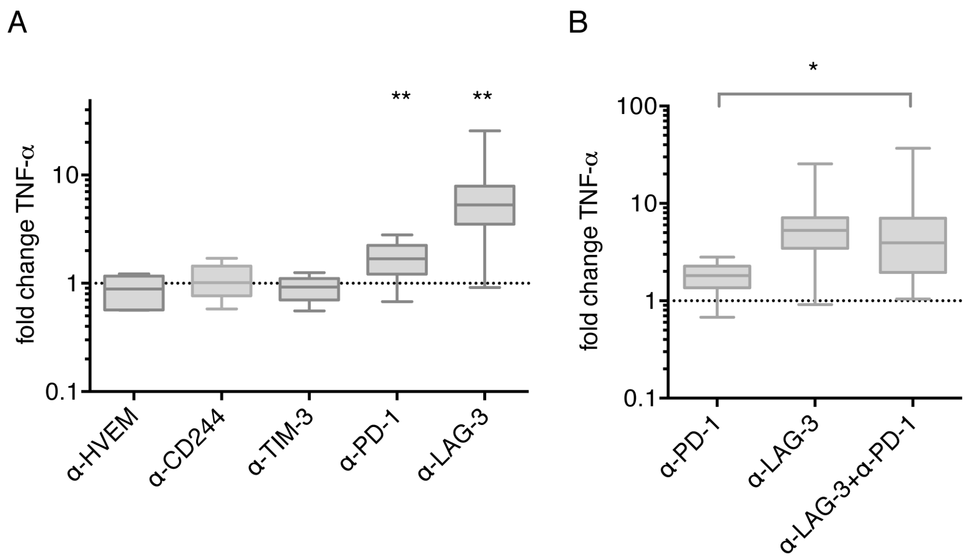

Figure S2. Effect of immune checkpoint blockade on TNF-α secretion of T cells after stimulation with TLR-3-DCs. $CD3^+$ T cells of 4–14 HDs were cocultured with autologous CEFT-pulsed TLR-3-DCs in the presence or absence of immune checkpoint blocking antibodies, either for individual antibodies (A) or in different combinations of α-PD-1 and α-LAG-3 antibodies (B). TNF-α secretion of $CD3^+$ T cells was determined by CBA assay, and the ratio between concentration with and without blocking antibody was calculated. All data are presented as box-and-whisker plots, and statistical significance was calculated against a fold change of 1.0. *, $p<0.05$; **, $p<0.01$.

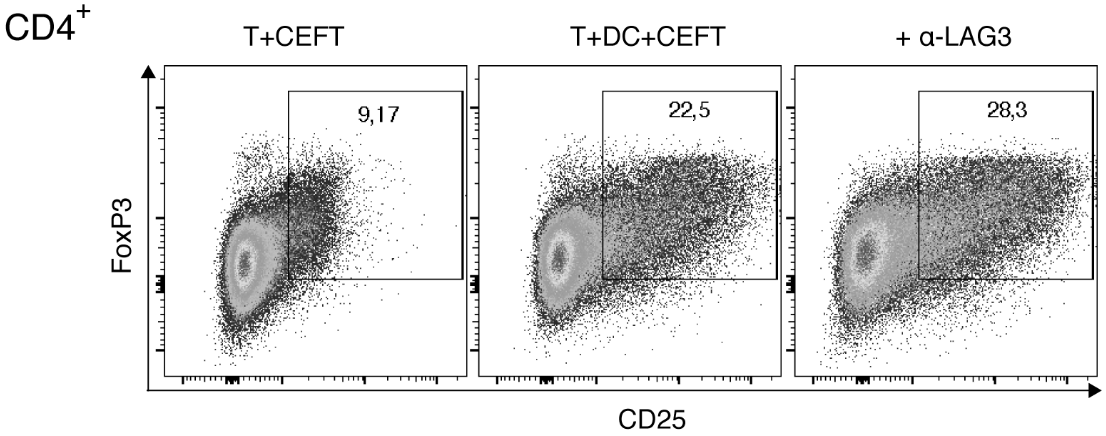

Figure S3. Effect of LAG-3 blockade on percentage of regulatory T cells after stimulation with TLR-3-DCs. $CD3^+$ T cells were cocultured with autologous CEFT-pulsed TLR-3-DCs in the presence or absence of α-LAG-3 antibody. Within the $CD4^+$ T cell population, co-expression of CD25 and FoxP3 was determined by flow cytometry. Data are shown for one representative donor, demonstrating a relative increase in percentage of regulatory T cells after LAG-3 blockade.

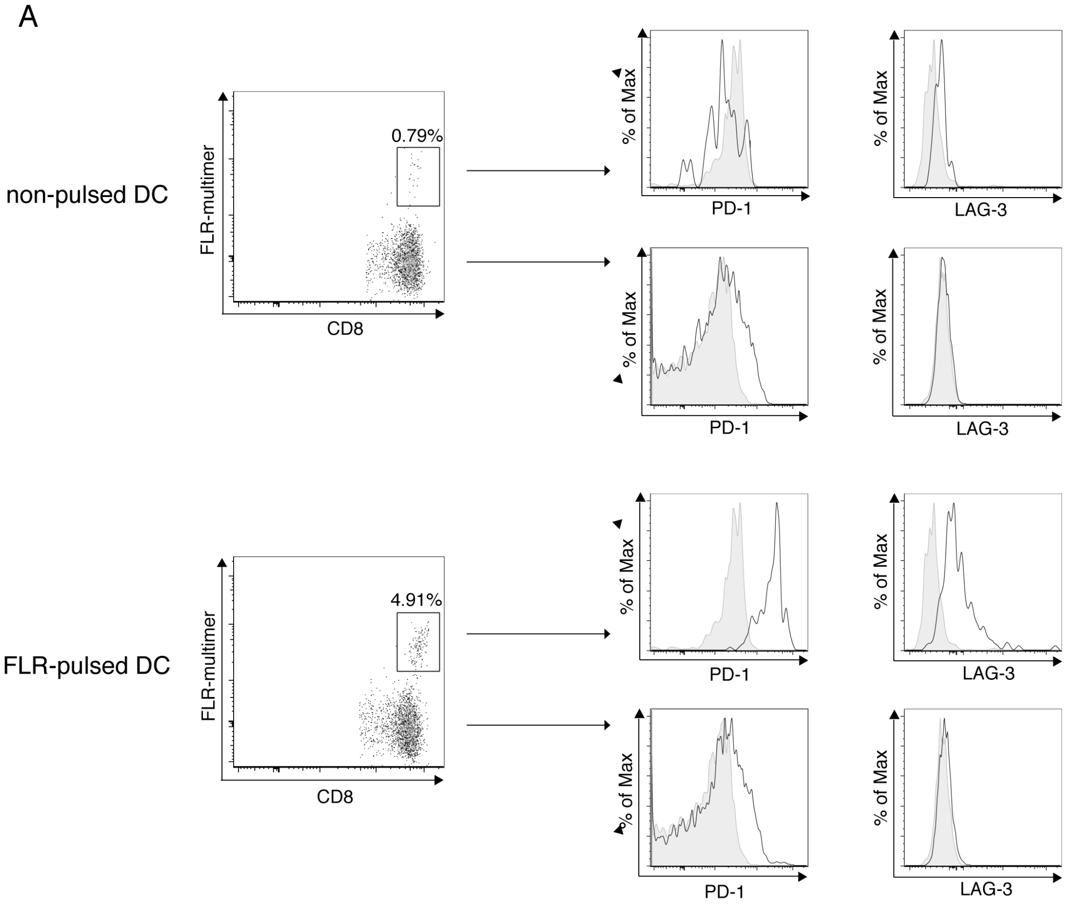
A
non-pulsed DC
FLR-pulsed DC
FLR-multimer
CD8
0.79%
4.91%
% of Max
PD-1
LAG-3

B

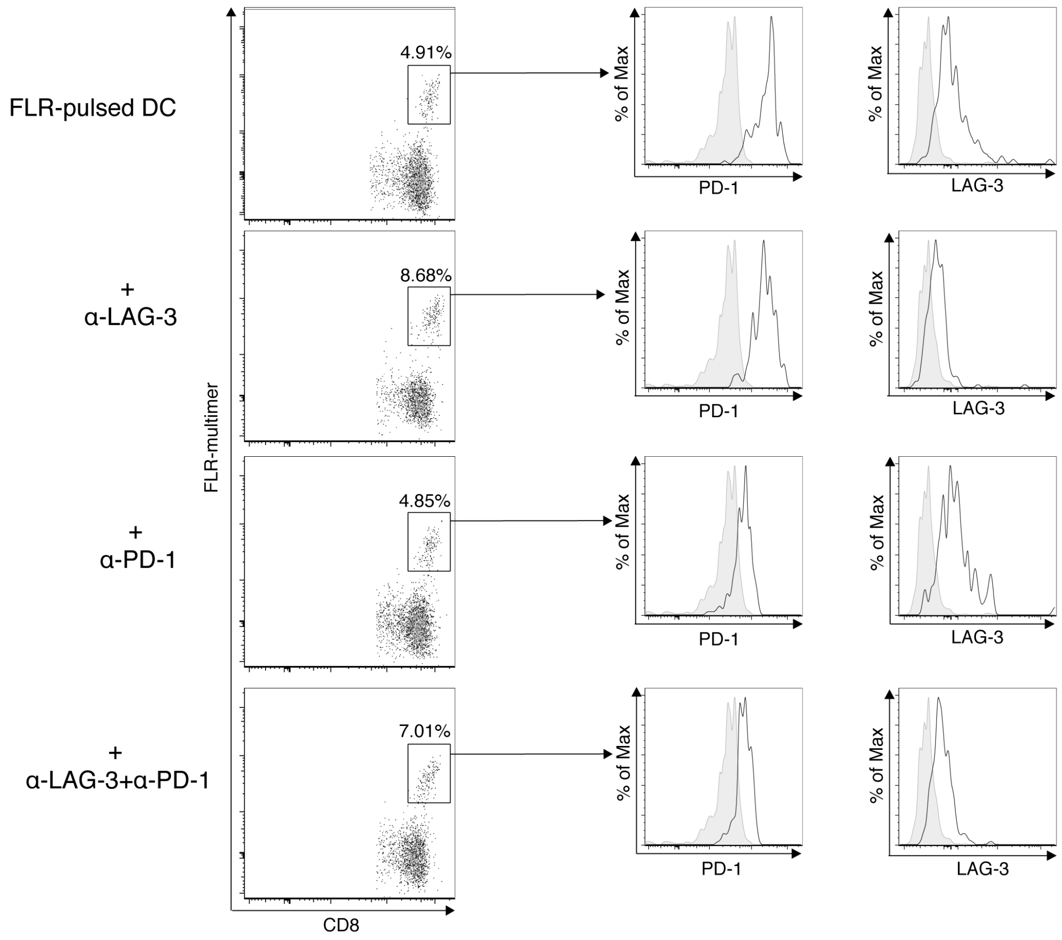

Figure S4. Competition between blocking antibodies and staining antibodies for LAG-3 and PD-1 on EBV antigen-specific T cells after stimulation with TLR-3-DCs. DCs were pulsed with FLR peptide and cocultured with autologous NAC in the presence or absence of α-PD-1 and α-LAG-3 blocking antibodies. Expression of PD-1 and LAG-3 positive cells within the FLR tetramer-positive cells of the $CD8^+$ T cell population was determined by flow cytometric measurements with respective staining antibodies. Data are shown for one representative donor.

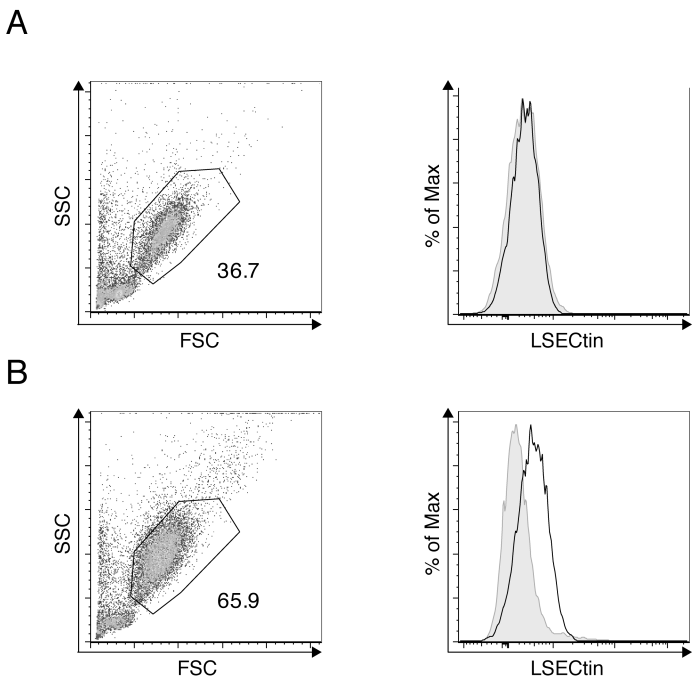

Figure S5. LSECtin expression of immature DCs and TLR-3-DCs. DCs were generated from PB of HDs, and surface marker expression was measured by flow cytometry. LSECtin expression of immature DCs (A), harvested on d2 of DC generation, before addition of maturation cocktail, and LSECtin expression of mature TLR-3-DCs (B), harvested on d3, after 24 hours with maturation cocktail, is shown for one representative donor.

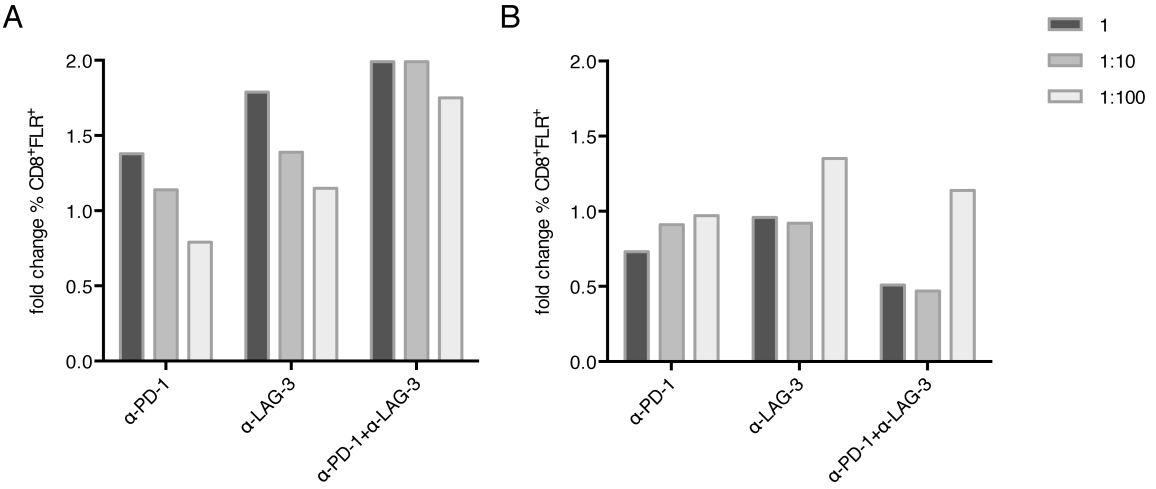

Figure S6. Effect of PD-1 and LAG-3 blockade on proliferation of EBV antigen-specific T cells after stimulation with PBMCs or TLR-3-DCs using different peptide concentrations. PBMCs (A) and TLR-3-DCs (B) were pulsed with FLR peptide in different dilutions and cocultured with NACs in the presence or absence of α-PD-1 and α-LAG-3 antibodies. The percentage of FLR tetramer-positive cells within the $CD8^+$ T cell population was determined by flow cytometry. Data for fold change to the condition without blocking antibody are shown for one representative donor.

A+D	$CD4^{+}$+DC^{+CEFT}	Ctrl	αHVEM	αCD244	αTIM3	αPD-1	αLAG-3	αPD-1+ αLAG-3
**HD1**	% divided	32.20	32.10	32.40	23.30	35.10		
**HD2**	% divided	10.80	10.60	10.40	11.00	13.30		
**HD3**	% divided	11.40	8.94	9.85	10.50	12.30		
**HD4**	% divided	13.60	15.10	13.90	12.10	15.30		
**HD5**	% divided	33.10					38.90	
**HD6**	% divided	16.90				17.20	37.20	
**HD7**	% divided	10.00				12.40	21.80	
**HD8**	% divided	21.70	3.30			21.40	50.10	49.80
**HD9**	% divided	42.70	13.50			44.70	54.20	51.60
**HD10**	% divided	15.70				39.00	37.50	47.10
**HD11**	% divided	30.10				34.10	35.70	38.00
**HD12**	% divided	50.00				51.00	59.80	70.80
**HD13**	% divided	37.00				36.50	42.80	43.10
**B+E**	**$CD8^{+}$+DC^{+CEFT}**	**Ctrl**	**αHVEM**	**αCD244**	**αTIM3**	**αPD-1**	**αLAG-3**	**αPD-1+ αLAG-3**
**HD15**	% divided	29.60	29.50	31.10	19.60	32.10		
**HD16**	% divided	11.50	10.90	11.80	12.00	14.10		
**HD17**	% divided	9.33	8.26	9.81	9.30	11.80		
**HD18**	% divided	12.70	11.80	13.40	13.20	15.10		
**HD19**	% divided	35.10					43.70	
**HD20**	% divided	10.30				12.00	22.80	
**HD21**	% divided	10.90				13.80	24.20	
**HD22**	% divided	14.60	2.99			16.30	36.10	53.30
**HD23**	% divided	39.00	31.60			39.40	61.00	60.90
**HD24**	% divided	8.82				11.60	22.80	24.90
**HD25**	% divided	19.50				19.80	24.60	25.10
**HD26**	% divided	41.60				39.00	44.10	46.70
**HD27**	% divided	37.90				38.50	43.60	41.90
**C+F**	**$CD3^{+}$ +DC+CEFT IFNγ**	**Ctrl**	**αHVEM**	**αCD244**	**αTIM3**	**αPD-1**	**αLAG-3**	**αPD-1+ αLAG-3**
**HD29**	pg/mL	398.63	387.30	715.76	402.48	964.74		
**HD30**	pg/mL	1381.55	541.91	1229.15	1523.45	9228.35		
**HD31**	pg/mL	194.64	123.56	142.63	138.33	256.26		
**HD32**	pg/mL	313.29	172.33	268.29	177.56	483.00		
**HD33**	pg/mL	851.05	304.34	516.54	946.29	787.98		
**HD34**	pg/mL	3485.22				4411.02		
**HD35**	pg/mL	594.75					2846.31	
**HD36**	pg/mL	378.06				620.73	3666.30	
**HD37**	pg/mL	8291.48				28491.01	271494.80	
**HD38**	pg/mL	2009.66				2733.42	5047.71	5513.86
**HD39**	pg/mL	15948.00				10193.00	27906.00	39155.00
**HD40**	pg/mL	937.11				1370.81	9714.00	12249.00
**HD41**	pg/mL	62772.98				69980.57	793222.10	940013.10
**HD42**	pg/mL	704.90	777.35			1340.94	3522.41	3609.71
**HD43**	pg/mL	1922.88	2321.78			3813.53	5553.99	5483.80
**HD44**	pg/mL	989.40				958.00	1593.60	2145.30
**HD45**	pg/mL	1121.40				2089.00	1837.00	2488.20

Table S1. Effect of immune checkpoint blockade on proliferation and IFN-γ secretion of T cells after stimulation with TLR-3-DCs. Original data on which Figure 3 is based.

frontiers
